# AAV-Mediated Gene Delivery in Adult GM1-Gangliosidosis Mice Corrects Lysosomal Storage in CNS and Improves Survival

**DOI:** 10.1371/journal.pone.0013468

**Published:** 2010-10-18

**Authors:** Rena C. Baek, Marike L. D. Broekman, Stanley G. Leroy, Laryssa A. Tierney, Michael A. Sandberg, Alessandra d'Azzo, Thomas N. Seyfried, Miguel Sena-Esteves

**Affiliations:** 1 Biology Department, Boston College, Chestnut Hill, Massachusetts, United States of America; 2 Program in Neuroscience, Department of Neurology, Massachusetts General Hospital and Harvard Medical School, Charlestown, Massachusetts, United States of America; 3 Department of Pharmacology and Anatomy, Rudolf Magnus Institute of Neuroscience, University Medical Center Utrecht, Utrecht, The Netherlands; 4 Berman-Gund Laboratory for the Study of Retinal Degenerations, Harvard Medical School, Massachusetts Eye and Ear Infirmary, Boston, Massachusetts, United States of America; 5 Department of Genetics, St. Jude Children's Research Hospital, Memphis, Tennessee, United States of America; University of Florida, United States of America

## Abstract

**Background:**

GM1-gangliosidosis is a glycosphingolipid (GSL) lysosomal storage disease caused by a genetic deficiency of acid β-galactosidase (βgal), which results in the accumulation of GM1-ganglioside and its asialo-form (GA1) primarily in the CNS. Age of onset ranges from infancy to adulthood, and excessive ganglioside accumulation produces progressive neurodegeneration and psychomotor retardation in humans. Currently, there are no effective therapies for the treatment of GM1-gangliosidosis.

**Methodology/Principal Findings:**

In this study we examined the effect of thalamic infusion of AAV2/1-βgal vector in adult GM1 mice on enzyme distribution, activity, and GSL content in the CNS, motor behavior, and survival. Six to eight week-old GM1 mice received bilateral injections of AAV vector in the thalamus, or thalamus and deep cerebellar nuclei (DCN) with pre-determined endpoints at 1 and 4 months post-injection, and the humane endpoint, or 52 weeks of age. Enzyme activity was elevated throughout the CNS of AAV-treated GM1 mice and GSL storage nearly normalized in most structures analyzed, except in the spinal cord which showed ∼50% reduction compared to age-matched untreated GM1 mice spinal cord. Survival was significantly longer in AAV-treated GM1 mice (52 wks) than in untreated mice. However the motor performance of AAV-treated GM1 mice declined over time at a rate similar to that observed in untreated GM1 mice.

**Conclusions/Significance:**

Our studies show that the AAV-modified thalamus can be used as a ‘built-in’ central node network for widespread distribution of lysosomal enzymes in the mouse cerebrum. In addition, this study indicates that thalamic delivery of AAV vectors should be combined with additional targets to supply the cerebellum and spinal cord with therapeutic levels of enzyme necessary to achieve complete correction of the neurological phenotype in GM1 mice.

## Introduction

GM1-gangliosidosis is a neurodegenerative lysosomal storage disease caused by deficiency of acid β-galactosidase (βgal) [1] leading to progressive accumulation of GM1-ganglioside in the CNS [2]. Age of onset of the symptoms ranges from infancy to adulthood and the severity of the clinical manifestations mostly correlates with the levels of residual enzyme activity [3]. In the most severe form of this disease (Infantile or Type I) biochemical and neuropathological alterations have been documented in utero [4], [5]. Progressive neurologic deterioration, macular cherry red spot, facial dysmorphism, hepatosplenomegaly, generalized skeletal dysplasia and early death are common features of the disease [3]. Currently there is no effective treatment for GM1-gangliosidosis.

The available knockout mouse models replicate several clinical and biochemical features of infantile GM1-gangliosidosis with low levels of βgal activity (<4% of normal) and massive accumulation of GM1-ganglioside and GA1 glycosphingolipid throughout the CNS [6], [7]. The βGal^−/−^ (GM1) mice used in this study accumulate abnormal levels of GM1-ganglioside as early as post-natal day 5 [8], and reach several fold above normal by 3 months of age [6], [9]. This feature is associated with a progressively severe CNS condition characterized by tremor, ataxia, abnormal gait and ultimately paralysis of the hind limbs [6]. Studies on this mouse model identified previously unknown molecular pathways that are induced by GM1 accumulation and result in neuronal apoptosis and neurodegeneration [10], [11]. Defective lysosomal degradation of GM1 was found to provoke the redistribution of this ganglioside at the ER membranes, where it induces depletion of ER Ca^2+^ stores, and in turn activation of the unfolded protein response (UPR) and UPR-mediated apoptosis [11]. More recently it was shown that GM1 accumulates specifically in glycosphingolipid-enriched fractions (GEMs) of the mitochondria-associated ER membranes, the sites of apposition between ER and mitochondria. GM1 at the GEMs favors Ca^2+^ flux between these organelles, which results in mitochondrial Ca^2+^ overload and activation of the mitochondrial leg of apoptosis [10]. Neuronal apoptosis is accompanied by neuroinflammation with increased microglial activation, production of inflammatory cytokines, chemokines, and inflammatory cell infiltration [12], [13].

Currently there is no effective treatment for GM1-gangliosidosis in children, although numerous therapeutic modalities have been implemented in GM1 mice with somewhat encouraging results [13], [14].

Direct infusion of adeno-associated virus (AAV) vectors encoding lysosomal enzymes into the brain parenchyma has emerged as a viable strategy to create an *in situ* source of normal enzyme in the brain [15]. A major obstacle to translation of the promising results obtained in animal models is the number of injections that may be needed to achieve global distribution of enzyme throughout the human brain. Based on studies in α-mannosidosis cats, it has been estimated that 40–60 injections of AAV vector may be necessary to obtain global distribution of lysosomal enzymes in the infant brain [16]. This large number of injections makes the treatment extremely invasive and with obvious risks. Therefore, alternative strategies are needed.

Previously we have shown that infusion of an AAV vector encoding βgal into the cerebral lateral ventricles of neonatal GM1 mice led to complete enzymatic and neurochemical correction of the brain in these animals [17]. Since most human patients are only identified after birth and newborn mice are developmentally equivalent to a human fetus, we set out to investigate a therapeutic approach that would be more relevant to the situation in humans. Lysosomal enzymes are distributed in the CNS from vector-transduced cells by axonal transport [18], [19], [20], [21] (among other mechanisms). One approach that takes advantage of this property is to target gene delivery to specific structures axonally connected to many areas of the brain. AAV-mediated gene delivery to the ventral tegmental area, one such structure, leads to widespread distribution of therapeutic levels of a lysosomal enzyme throughout the mouse brain [22]. The thalamus as the major information relay center in the brain receives and processes sensory, motor, limbic and arousal input from various regions of the CNS and relays the information to multiple brain structures including the cerebral cortex. It is one of the most interconnected structures in the brain, and thus an appealing target for gene delivery to achieve widespread distribution of lysosomal enzymes via axonal transport. Also AAV-mediated gene transfer to deep cerebellar nuclei (DCN) appears to be an effective approach to deliver lysosomal enzymes and growth factors to cerebellum and spinal cord [23], [24]. Here GM1 mice received bilateral infusions of AAV vector encoding mouse βgal into the thalamus or thalamus and deep cerebellar nuclei at six to eight weeks of age and we analyzed the effects of this treatment on neurochemistry, motor performance over time and survival. Our results show that AAV-mediated gene delivery in adult GM1 mice increased βgal activity and reduced GM1-ganglioside levels to nearly normal in the brain, but only ∼50% in the spinal cord. Although the motor performance of AAV-treated GM1 mice remained comparable to that of untreated GM1 mice, their survival was significantly extended with mice receiving bilateral injections in thalamus and DCN surviving until 12 months of age.

## Materials and Methods

### Mice

GM1-gangliosidosis (GM1) mice were obtained from Dr. Alessandra d'Azzo (St. Jude Children's Research Hospital, Memphis, TN) and have been described previously [6].

### AAV Vector Design and Preparation

The design and production of AAV2/1-CBA-βgal vector carrying the mouse lysosomal acid beta-galactosidase (βgal) cDNA under the CBA promoter, which is comprised of the CMV immediate-early enhancer fused to the chicken beta-actin promoter, was described previously [17]. The plasmid pAAV-ApoE4hAAT-βgal-W was constructed by replacing the CBA promoter in the plasmid pAAV-CBA-βgal-W [17] with the hybrid ApoE4/hAAT liver specific promoter (human alpha-1 antitrypsin promoter fused to 4 copies of the apolipoprotein A enhancer [25] (kindly provided by Dr. Valder Arruda, The Children's Hospital of Philadelphia, Philadelphia, PA). The AAV2/rh8-ApoE4hAAT-βgal vector was prepared as described [26]. All vectors used in this study carry the woodchuck hepatitis virus post-transcriptional regulatory element (WPRE).

### Delivery of AAV vector to the brain

Six to eight week-old GM1 mice were anesthetized by intraperitoneal injection of ketamine (125 mg/kg) and xylazine (12.5 mg/kg) in 0.9% saline, and placed in a small animal stereotaxic frame (Stoelting, Wood Dale, IL). An incision was made over the skull, the periosteum removed and a burr hole was made at the appropriate stereotaxic coordinates using a high-speed drill (Dremel, Racine, WI). The noncompliant infusion system used in these experiments for delivery of AAV vector was assembled using a Harvard 22 syringe pump (Harvard Apparatus, Holliston, MA) to drive a gas-tight Hamilton Syringe (Hamilton, Reno, NV) attached to a 33-gauge steel needle (Hamilton) via 1/16″×0.020″ ID PEEK tubing (Alltech, Deerfield, IL) and Luer adapters (Amersham Biosciences). First the syringe and tubing were filled with sterile mineral oil and then vector stock was withdrawn into the needle and line. The needle assembly (needle+Luer adapters) was fixed to the arm of the stereotaxic frame. AAV2/1-CBA-βgal vector was infused (1 µl at 0.2 µl/min) into the left thalamus (AP −2.0 mm, ML −1.5 mm relative to bregma, and DV −2.5 mm from the brain surface). In subsequent therapeutic efficacy experiments the AAV2/1-CBA-βgal vector was infused (0.2 µl/min) bilaterally into the thalamus at two depths (AP −2.0 mm, ML +/−1.5 mm relative to bregma; and −3.5 and −2.5 mm from the brain surface; 1 µl per depth) (total dose per mouse = 4.8×10^10^ gc), or bilaterally into the thalamus (as above) and deep cerebellar nuclei (AP: −6.3 mm; ML: +/−1.5 mm; DV −2.0 mm; 1 µl per side) (total dose per mouse = 7.2×10^10^ gc). In the PBS control group, age matched GM1 mice received bilateral infusion of PBS into the thalamus and deep cerebellar nuclei (same infusion rate and volume as above). The needle was left in place for 2.5 min after the injection was finished and then retracted halfway and left in place for an additional 2.5 min before complete withdrawal. The incision was closed with surgical staples, or colloidin, and the animal was allowed to recover completely before being returned to the holding room.

### Behavioral Testing

#### Rotarod test

A rotarod apparatus, consisting of a knurled dowel fixed 10 cm above bedding was used to measure motor coordination and balance as previously described [27]. After a 3-day pretrial training period, mice were assessed for motor behavior at 1, 2.5, 4, and 6 months post injection. Mice were placed on the rotating dowel at 20 rpm, indicating the start time for the trial. A 30-second interval was allowed between the two trials at the given speed. The maximum time allowed on the bar for each trial was 60 seconds. The trial was terminated when the mouse fell off the bar or at 60 seconds.

#### Open-field test

Locomotor activity and rearing events in the mice were assessed using the SmartFrame Cage Rack System (Kinder Scientific, San Diego, CA). Infrared beams along the frame of the system track mouse movement in the cage with respect to location, distance, and rearing capabilities. Mice were placed in the center of the open-field apparatus and behavior was measured for 15 minutes. The data was analyzed using the MotorMonitor software (Kinder Scientific, San Diego, CA). Locomotor activity was measured as the distance traveled (in inches) and rearing events were measured as the number of times the mouse stood on its hind legs. Comparisons were narrowed to the first 5 minutes when we found significant differences between untreated GM1 and HZ mice, as previously shown [13].

### Visual evoked potentials

Visual evoked potentials (VEPs) were recorded in AAV-treated GM1 mice (AAV-T and AAV-TC groups; n = 3 for each group) at 9–10 months of age. The VEPs were also recorded from untreated heterozygote (n = 2) and GM1 (n = 4) mice at 10 months and 7–8 months of age, respectively, according to previously described methods [28]. Briefly, the mice were dark-adapted overnight and then anesthetized. The left pupil was dilated and responses were elicited with 10-µs full-field flashes of white light presented every second at 3.4 log ft.L. VEPs were monitored with subdermal electrodes in the scalp over the visual cortex as the positive electrode and over the frontal cortex as the reference. The responses were collected as previously described [28]. The consecutive waveforms were averaged (n = 100) after suppressing the heart-beat artifact with an adjustable low-pass digital filter (cut-off at 50–70 Hz) and rejecting waveforms containing movement artifacts.

All animal experiments were carried out with ethical committee approval in accordance with the National Institutes of Health Guide for the Care and Use of Laboratory Animals and were approved by the Institutional Animal Care Committees at the Massachusetts General Hospital (Approved Protocol #: 2003N000343/3) and Boston College (Approved Protocol #: 2009-014-01).

### Tissue Preparation

Mice were killed by CO_2_ asphyxiation at 1 or 4 months post-injection or at the humane endpoint defined by >20% loss in maximal body weight. The brains were harvested at 1 and 4 months post injection and at the humane endpoint. The left hemisphere of the brain was embedded in tissue freezing medium (Triangle Biomedical Sciences, Durham, NC) and rapidly frozen in a 2-methylbutane/dry-ice bath. Consecutive 20-µm thick coronal cryosections were prepared and stored at −80°C. One series of frozen sections representing the entire brain from AAV-treated GM1 mouse, or control non-injected GM1 mice was fixed for 10 min in 0.25% glutaraldehyde in phosphate buffered saline (PBS) at room temperature followed by two washes in PBS. Sections were stained for βgal using X-gal solution [5 mM K_4_Fe(CN)_6_, 5 mM K_3_Fe(CN)_6_, 2 mM MgCl_2_, 1 mg/ml 5-bromo-4-chloro-3-indolyl-D-galactosidase (X-gal) in PBS, pH 5.0 as described [17].

The right hemisphere of the brain was grossly dissected into cortex, cerebellum, brainstem plus sub-cortical structures (Bs+ScS; subcortical structures included midbrain, thalamus, hypothalamus, hippocampus and striatum), and rapidly frozen on dry ice. The right hemisphere was used for analysis of βgal enzymatic activity and lipids. In addition, the spinal cord was divided into 0.5 cm transverse sections. Alternating sections were collected and analyzed for GSL storage. The values obtained in untreated and PBS-treated GM1 mice were indistinguishable from each other and as a result, were pooled together.

### β-Galactosidase Assay

Total β-galactosidase activity was determined using 4-methylumbelliferyl-β-D-galactopyranoside as the synthetic fluorogenic substrate, specific for β-galactosidase as previously described [9]. Total β-galactosidase activity was determined by measuring the release of 4-methylumbelliferone at excitation 360 nm, emission 460 nm on a SpectraMax M5 plate reader (Molecular Devices, Sunnyvale, CA) and normalized to total protein concentration.

### Isolation and Purification of Glycosphingolipids

#### Total Lipid Extraction

Total lipids were extracted as previously described [29]. Briefly, frozen brain samples were lyophilized and the dry weights were measured. Total lipids were extracted from the lyophilized brain samples in a solution of chloroform (CHCl_3_)∶methanol (CH_3_OH) and distilled water (dH_2_O). The samples were placed on a magnetic stirrer at room temperature for at least 8 hours and then centrifuged for 15 minutes at 1200 g. The supernatant was collected and the pellet was washed with CHCl_3_∶CH_3_OH (1∶1 by volume) and centrifuged as before. The combined supernatants were converted to a CHCl_3_∶CH_3_OH∶dH_2_O ratio of 30∶60∶8 (solvent A).

#### Column Chromatography

Neutral and acidic lipids were separated using DEAE-Sephadex (A-25, Pharmacia Biotech, Upsala, Sweden) column chromatography as previously described [30]. DEAE-Sephadex was prepared in bulk by washing the resin three times with solvent B (CHCl_3_∶CH_3_OH∶0.8 M Na acetate, 30∶60∶8 by volume), equilibrating in solvent B overnight, followed by washing three times with solvent A (CHCl_3_∶CH_3_OH∶dH_2_0, 30∶60∶8 by volume) until neutral. The total lipid extract, suspended in solvent A, was applied to a DEAE-Sephadex column. The column was washed twice with solvent A and the entire neutral lipid fraction, consisting of the initial eluent plus washes, was collected. This fraction contained cholesterol, phosphatidylcholine, phosphatidylethanolamine and plasmologens, sphingomyelin, and neutral GSLs to include cerebrosides and asialo-GM2 (GA2). Next, acidic lipids were eluted from the column with solvent B.

#### Ganglioside Purification

The acidic lipid fraction containing gangliosides was dried by rotary evaporation and transferred to a 15 mL graduated conical glass tube. Water was added and the mixture was inverted, vortexed, and centrifuged for about 15 minutes at 1200×g to partition gangliosides into the upper phase [31]. The upper aqueous phase was removed and the lower organic phase was washed once with Folch ‘pure solvent upper phase’ (CHCl_3_∶CH_3_OH∶dH_2_0, 3∶48∶47 by volume).

#### Resorcinol Assay

An aliquot of the ganglioside fraction (Folch upper phase) was evaporated and analyzed for sialic acid content using a modified resorcinol assay [32]. *N*-acetylneuraminic acid (Sigma # A9646, St. Louis, MO, USA) (1, 3, and 6 µg) was used as an external standard. Samples were dissolved in 1 mL of resorcinol reagent (40 mL concentrated HCl, 0.125 mL 0.1 M copper sulfate, 5 mL 2% resorcinol stock, brought to 50 mL with distilled water)∶dH_2_O (1∶1 by volume), boiled for 17 minutes, and then cooled in an ice bath. Butyl acetate∶1-butanol (1.5 mL, 85∶15 by volume) was then added to each sample, and the samples were vortexed and centrifuged at 1200×g. The supernatant was then carefully removed and analyzed in crystal cuvettes at 580 nm in the Shimadzu UV-1601 UV-visible spectrophotometer (Shimadzu, Kyoto, Japan).

#### Base Treatment and Desalting

After removing aliquots for the resorcinol assay, the ganglioside fraction was evaporated under a stream of nitrogen and treated with mild base (1 mL of 0.5 M NaOH) in a shaking water bath at 37°C for 1.5 hours. Base and salts were separated from the gangliosides using a modification of a previously described method [33]. Briefly, the sample was applied to a C18 reverse-phase Bond Elute column (Varian, Harbor City, CA, USA) and then the column was washed with dH_2_O to remove salts. Gangliosides were eluted from the column with CH_3_OH and then followed CHCl_3_∶CH_3_OH (1∶1 by volume). Samples were evaporated under nitrogen, re-susupended in CHCl_3_∶CH_3_OH (1∶1 by volume), and then stored at 4°C.

Acidic Phospholipid Purification - After the ganglioside fraction (Folch upper phase) was transferred, the acidic phospholipid fraction (Folch lower phase) was evaporated under a stream of nitrogen and resuspended in CHCl_3_∶CH_3_OH (1∶1 by volume). This fraction contained fatty acids, cardiolipin, phosphatidylserine, phosphatidylinositol, sulfatides.

#### Neutral Lipid Purification

Neutral lipids were dried by rotary evaporation and resuspended in CHCl_3_∶CH_3_OH (2∶1 by volume). To further purify GA1, an aliquot of the neutral lipid fraction was evaporated under a stream of nitrogen, base treated with 1 N NaOH, and Folch partitioned as described previously [8], [30]. The Folch lower phase containing GA1 was evaporated under a stream of nitrogen and re-suspended in CHCl_3_∶CH_3_OH (2∶1 by volume).

### Analysis of Glycosphingolipids by HPTLC

All lipids were analyzed qualitatively by high-performance thin-layer chromatogram (HPTLC) according to previously described methods [8], [30]. To enhance precision, an internal standard (oleoyl alcohol) was added to the neutral and acidic lipid standards and samples as previously described. Purified lipid standards were either purchased from Matreya Inc. (Pleasant Gap, PA, USA), Sigma (St. Louis, MO, USA), or were a gift from Dr. Robert Yu (Medical College of Georgia, Augusta, GA, USA). Lipids were spotted on 10×20 cm Silica gel 60 HPTLC plates (E. Merck, Darmstadt, Germany) using a Camag Linomat V semi-automatic TLC spotter (Camag Scientific Inc., Wilmington, NC, USA).

For gangliosides and GA1, the HPTLC plates were developed by a single ascending run with CHCl_3_∶CH_3_OH∶dH_2_O (55∶45∶10 by volume for gangliosides and 65∶35∶8 by volume for GA2) containing 0.02% CaCl_2_2H_2_O. The plates were sprayed with either resorcinol-HCl or orcinol-H_2_SO_4_ reagent and heated on an aluminium block heater at 105°C for approximately 30 minutes to visualize gangliosides or GA1, respectively [32].

For neutral and acidic phospholipids, the plates were developed to a height of either 4.5 cm or 6 cm, respectively with chloroform: methanol: acetic acid: formic acid: water (35∶15∶6∶2∶1 by volume), and then both were developed to the top with hexanes: diisopropyl ether: acetic acid (65∶35∶2 by volume) as previously described [34]. Neutral and acidic phospholipids were visualized by charring with 3% cupric acetate in 8% phosphoric acid solution, followed by heating in an oven at 165°C for 7 minutes.

The percentage distribution and density of individual bands was determined as previously described [8]. Briefly, the HPTLC plates were scanned on a Personal Densitometer SI with ImageQuant software (Molecular Dynamics) or on a ScanMaker 4800 with ScanWizard5 V7.00 software (Microtek). The total brain ganglioside distribution was normalized to 100% and the percentage distribution values were used to calculate sialic acid concentration of individual gangliosides as we previously described [35]. The density value for GA1 was fit to a standard curve of known lipid concentration and used to calculate concentration. For the neutral and the acidic phospholipids, each lipid was normalized to an internal standard (oleoyl alcohol) and its concentration was quantified using a standard curve of each respective lipid. All brain lipid concentrations are expressed as mg/100 mg dry weight.

### Real-Time PCR

RNA was isolated from cerebrum, cerebellum, brainstem plus sub-cortical structures (Bs+ScS), and spinal cord using Trizol reagent (Invitrogen, Carlsbad, CA), and first-strand cDNA synthesis was performed using Omniscript reverse transcriptase (Qiagen, Valencia, CA) according to manufacturers' instructions. Real-time PCR analysis of disease marker gene expression was performed using Taqman Gene Expression Assays (Applied Biosystems, Foster City, CA.) for TNFα, FAS, MIP-1α,, MHC class II, F4/80, and GAPDH as housekeeping gene. PCR was performed on a 7500 Fast Real-time PCR system (Applied Biosystems) in Fast Mode. Gene expression levels were normalized to GAPDH (ΔCt) and then compared to heterozygote (HZ) levels using the formula 2^ΔΔCt^ to calculate fold over HZ level for each gene. Error associated with fold change was calculated using the following formula [36]: 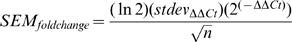.

### Statistical Analysis

Data were analyzed by one-way analysis of variance or Student's t-test to calculate statistical significance between untreated GM1 mice, AAV-treated GM1 mice, and control HZ mice using Statview 5.0, or Microsoft Excel software.

## Results

### Thalamic infusion of AAV vector in adult GM1 mice results in widespread distribution of βgal in the brain

**Figure 1:**
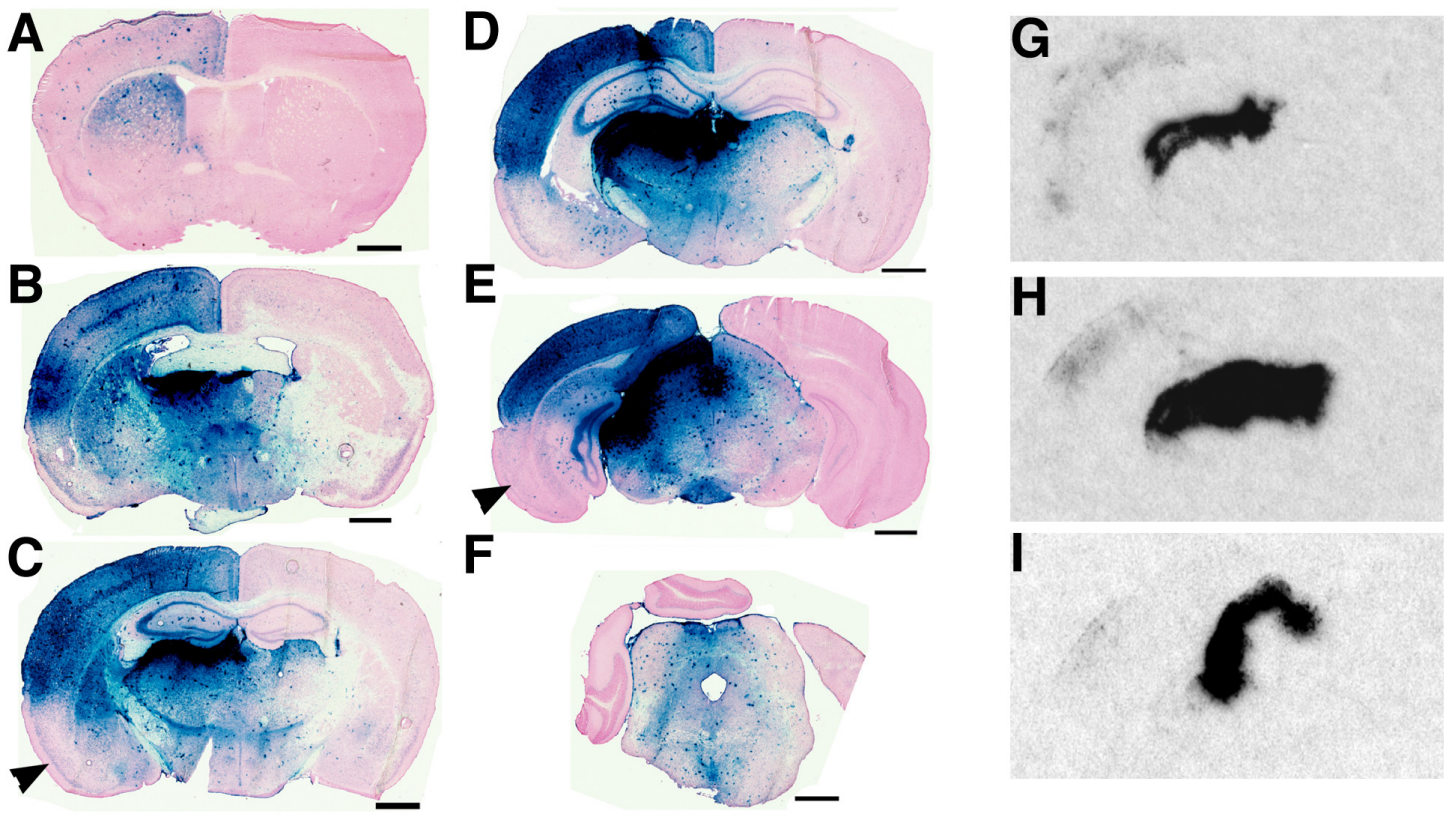
Distribution of βgal in the brain after AAV1-mediated thalamic gene delivery. One µl of AAV2/1-CBA-mβgal vector (4.12×10^13^ gc/ml) was injected into the left thalamus of 6–8 week-old GM1 mice. (**A–F**) One month later the brain was analyzed for βgal distribution by X-gal histochemical staining. βgal staining was evident throughout the ipsilateral hemisphere. Arrowhead in (**C**) indicates the ipsilateral perirhinal and piriform cortices where bgal staining is less intense than elsewhere in the cortex in the same coronal plane. (**G–I**) Detection of vector mRNA by radioactive *in situ* hybridization. (**G**), (**H**), (**I**) show the signal detected in tissue sections adjacent to those stained for βgal activity with X-gal shown in (**C**), (**D**), and (**E**), respectively. Arrowheads in (**C**), and (**E**) indicate the perirhinal, piriform and entorhinal cortices. Scale bars in (**A–F**) represent 1mm.

We injected 1 µl of AAV2/1-CBA-mβgal vector (4.12×10^13^ gc/ml) into the left thalamus of 6–8 week-old GM1 mice and one month later analyzed lysosomal acid beta-galactosidase distribution in the brain by X-gal histochemical staining of tissue sections (Fig. 1). The highest staining intensity (dark blue) was observed in dorsal and lateral thalamus, but intense βgal staining was observed in retrosplenial, visual, somatosensory, and auditory cortices. Interestingly there was a clear boundary of enzyme distribution in the cortex with lower βgal staining in perirhinal, piriform and entorhinal cortices (arrowheads in Fig. 1). In anterior regions of the brain (Fig. 1A) we found that βgal staining declined from the cingulate cortex to dorsolateral regions of the cortex (motor and somatosensory). In the striatum the most intense βgal staining was associated with the dorsal region (Fig. 1A). ISH analysis showed that AAV-transduced cells were present in dorsal and lateral thalamic nuclei only (Fig. 1G–I) and the signal intensity correlated with areas in the brain (thalamus) that reacted most strongly with the X-gal substrate (Fig. 1A–F). Some faint ISH signal was also apparent in the ipsilateral cortex.

### Intraparenchymal infusion of AAV vector in adult GM1 mice results in sustained βgal expression throughout the CNS

Disease progression in the GM1 mouse model is associated with microgliosis, production of inflammatory cytokines and chemokines, up-regulation of inflammatory markers, and inflammatory cell infiltration [12], [13]. Beside the thalamus and hypothalamus, these alterations are also detected prominently in the spinal cord and cerebellum. As delivery of lysosomal enzymes to these structures can be achieved by injection of AAV vectors into the deep cerebellar nuclei [23], we included a group of GM1 mice injected bilaterally in the thalamus and deep cerebellar nuclei. Also since GM1 mice are βgal mRNA negative [6], we tested a strategy previously used in the Niemann-Pick mouse to prevent confounding effects of a potential immune response against AAV-produced enzyme in the brain [37]. This approach consists of an intravenous infusion of a second AAV vector encoding the respective enzyme from a liver-specific promoter prior to intracranial injection of the test AAV vector encoding the same enzyme.

We injected 6–8 week-old GM1 mice intravenously (i.v.) with 3×10^11^ gc of AAV2/rh8-ApoE4hAAT-βgal vector via the tail vein. Two weeks later we performed bilateral injections of AAV2/1-CBA-βgal vector (1.2×10^13^ gc/mL) into the thalamus (AAV-T group: n = 23; Total dose = 4.8×10^10^ gc); or bilateral injections into the thalamus and deep cerebellar nuclei (AAV-TC group: n = 22; Total dose = 7.2×10^10^ gc). As controls we included age matched GM1 mice infused i.v. with AAV2/rh8-ApoE4hAAT-βgal followed by intracranial injection of PBS in thalamus and cerebellum (n = 22), age matched untreated GM1 (n = 22), and HZ (n = 24) mice. Mice were sacrificed at 1 and 4 months post-injection, or at the humane endpoint defined by >20% loss in maximum body weight achieved by each mouse. βgal activity in the serum of GM1 mice at one month after tail vein injection was 25% of the level measured in the serum of age-matched naïve HZ mice.

**Figure 2:**
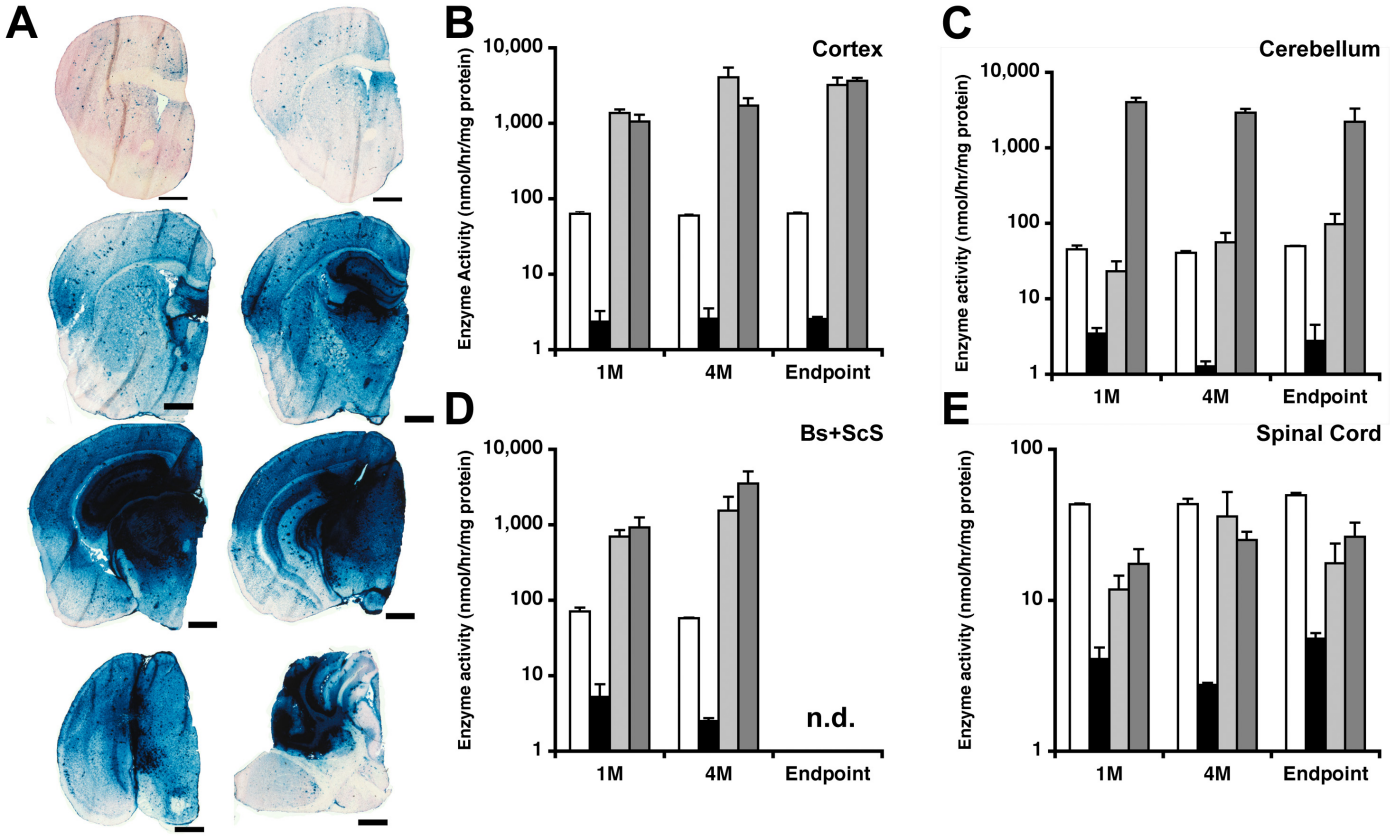
β-galactosidase distribution in CNS of GM1-gangliosidosis mice after intraparenchymal infusion of AAV2/1-βgal vector. Six to eight week-old GM1 mice received bilateral injections of AAV2/1-βgal vector (1.2×10^13^ gc/ml) into the thalamus (AAV-T; light gray bars), or thalamus and deep cerebellar nuclei (AAV-TC; dark gray bars). Age matched heterozygote (white bars) and untreated GM1 mice (black bars) were used as controls. Mice were sacrificed at 1, and 4 months post-infusion and at the humane endpoint (Endpoint). (**A**) The left brain hemisphere was used for histological analysis of βgal distribution by X-gal staining at pH 5.0. Representative sections from an AAV-TC GM1 mouse sacrificed at 4 months post-injection are shown. Scale bar = 1 mm. βgal activity was determined by 4MU assay in (**B**) cortex, (**C**) cerebellum, (**D**) brainstem+subcortical structures (Bs+ScS), and (**E**) spinal cord at 1 (1M) and 4 (4M) months post-injection, and at the humane endpoint (Endpoint). Error bars correspond to standard error of the mean. n.d. – not determined.

βgal enzymatic activity in the CNS of treated and control mice was analyzed both qualitatively and quantitatively (Fig. 2). X-gal staining of brain sections showed widespread βgal activity throughout the brain in both groups of AAV-treated GM1 mice (Fig. 2A). The βgal distribution pattern in the brain remained essentially unchanged over time (data not shown). In the cerebellum intense βgal staining was observed only in AAV-TC GM1 mice (Fig. 2A). βgal activity against 4MU substrate was more than 10-fold higher than the enzyme levels in HZ mice (white bars in Fig. 2) in cortex and brainstem+sub-cortical structures (Bs+ScS) from both groups of AAV-injected GM1 mice (gray bars in Figs. 2B, C). βgal activity in the cerebellum of AAV-T GM1 mice was restored to HZ values by 4 months post-injection and showed a slight increase at the final endpoint (light gray bars, Fig. 2D). In contrast, in the cerebellum of AAV-TC GM1 mice βgal activity was >10-fold higher than in HZ cerebellum at 1 month post-injection and remained elevated for the duration of the experiment (dark gray bars, Fig. 2D). In AAV-T GM1 mice, βgal activity in the spinal cord reached a maximum of 82% of HZ levels at 4 months post-injection and then declined to ∼35% at the final endpoint (light gray bars, Fig. 2E). In AAV-TC GM1 mice, βgal activity in the spinal cord remained essentially stable after 4 months post-infusion at ∼53% of HZ level (dark gray bars, Fig. 2E). The βgal activity in the CNS of PBS-injected GM1 mice was indistinguishable from that in age matched untreated GM1 controls (black bars, Fig. 2) at about 1–2% of HZ levels.

### Sustained reduction in GM1-ganglioside content in the CNS of AAV-treated GM1 mice

**Figure 3:**
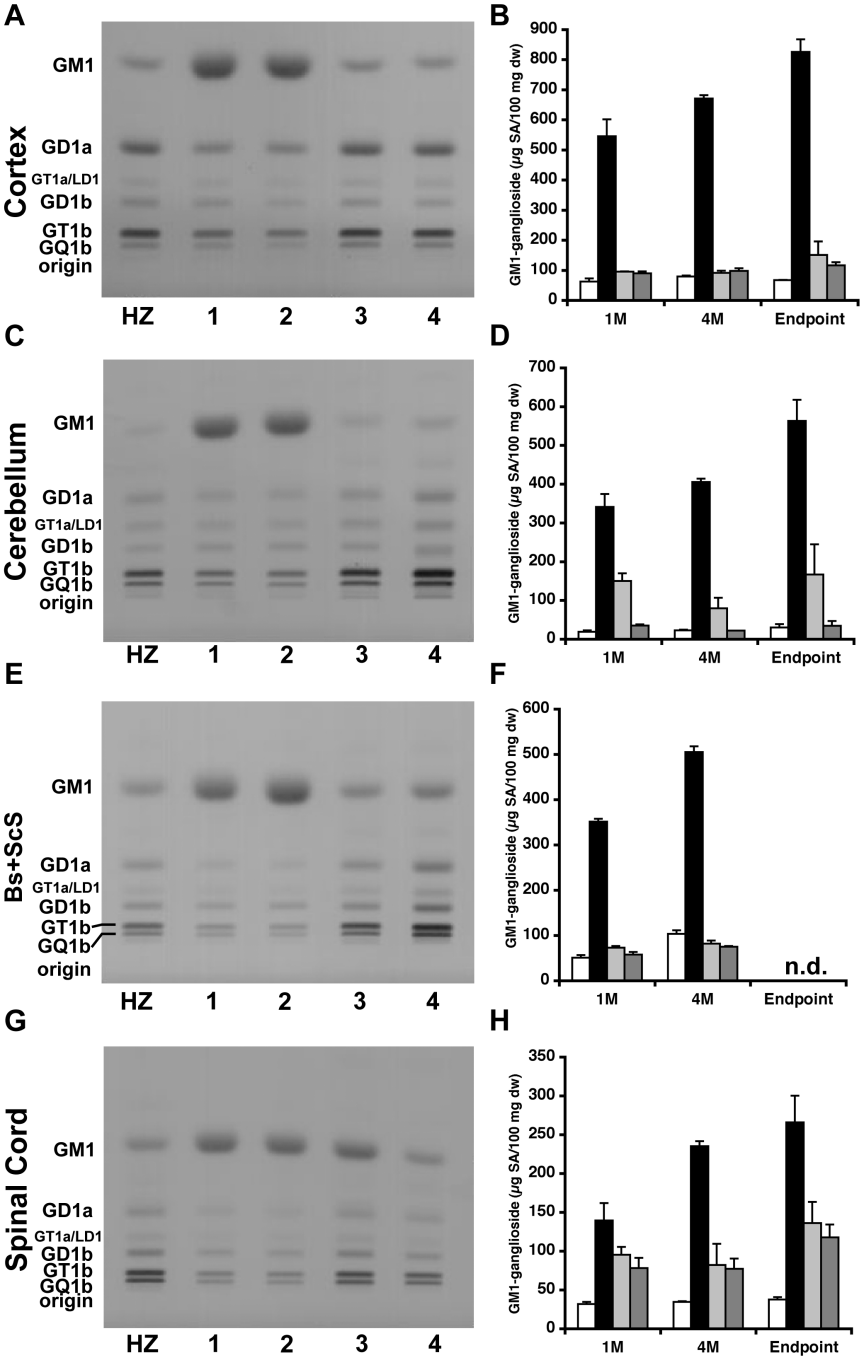
Biochemical quantification of GM1-ganglioside content in the CNS of AAV-treated GM1 mice by HPTLC. Gangliosides were isolated from (**A, B**) cortex, (**B, C**) cerebellum, (**D, E**) brainstem+subcortical structures (Bs+ScS), and (**F, G**) spinal cord of heterozygote (HZ, white bars), untreated GM1 (1, black bars), PBS-treated GM1 (2), AAV-T GM1 (3, light gray bars), and AAV-TC GM1 (4, dark gray bars) mice. (**A, C, E, G**) Representative chromatograms showing the qualitative distribution of gangliosides at 4 months post-injection. The amount of gangliosides spotted per lane was approximately 1.5 µg sialic acid. The individual gangliosides were labeled according to the nomenclature system of Svennerholm (left side of the chromatograms) [32]. (**B, D, F, H**) Mean concentration of GM1-ganglioside in each region of the CNS at 1 month (1M), 4 months (4M) post-injection, and at the humane or experimental endpoint (Endpoint) are shown. Error bars represent 1 SEM. n.d. – not determined.

We next analyzed the same CNS regions from all groups of mice and time points for the distribution of individual gangliosides by high-performance thin layer chromatography (HPTLC) (Fig. 3). The qualitative and quantitative distributions of individual gangliosides in cortex, brain stem and subcortical structures (Bs+ScS), cerebellum, and spinal cord at 4 months post-injection are shown in Fig. 3A–H and Tables S1, S2, S3, S4, S5, S6, S7, S8, S9, S10, S11. As expected, GM1-ganglioside content in untreated or PBS-injected GM1 mice increased with age in all regions analyzed (black bars in Fig. 3A, C, E, G – lane 2). In contrast, GM1-ganglioside levels were dramatically reduced in both groups of AAV-treated GM1 mice (light and dark gray bars in Fig. 3). In AAV-T GM1 mice reductions in GM1-ganglioside content ranged from a minimum of 31.6% (spinal cord at 1 month post-injection) to a maximum of 86.4% (cerebral cortex at 4 months post-injection) compared to the levels measured for the same structures in age-matched untreated GM1 mice. In AAV-TC GM1 mice GM1-ganglioside content was reduced from a minimum of 44% (spinal cord at 1 month post-injection) to a maximum of 93.5% (cerebellum at 4 months post-injection) relative to the levels measured for the same structures in age-matched untreated GM1 mice. However, GM1-ganglioside levels in both groups of AAV-treated mice remained consistently higher than levels in HZ mice, with the exception of Bs+ScS at 4 months post-injection in both groups, and the cerebellum in AAV-TC GM1 mice (Fig. 3F and Tables S5, S7, and S8). By the humane (AAV-T) or experimental (AAV-TC) endpoint the GM1-ganglioside content in all structures analyzed was higher than normal levels in control HZ mice (Fig. 3B, D, H – Endpoint light and dark gray bars). Additional injection of AAV vector into the deep cerebellar nuclei had a significant impact on the biochemical outcome in the cerebellum, but only a marginal effect on the spinal cord GM1-ganglioside content compared to thalamic injection alone.

An interesting observation was made when analyzing the distribution of individual gangliosides following intracranial AAV treatment. At 1 month post-injection, GM2- and GM3-gangliosides were detected by HPTLC in all regions analyzed (Tables S1, S4, S6, S9). However, at 4 months post-injection and at endpoint, GM2- and GM3-gangliosides were no longer present.

Ganglioside GD3, a minor glycolipid in adult mouse CNS [38], was absent in the cortex of all mice (Tables S1, S2, S3), but present in Bs+ScS, cerebellum, and spinal cord (Tables S4, S5, S6, S7, S8, S9, S10, S11). This ganglioside was decreased in Bs+ScS, cerebellum, and spinal cord of untreated GM1 mice compared to age-matched naïve HZ mice (Tables S4, S5, S6, S7, S8, S9, S10, S11). In AAV-T and AAV-TC mice the ganglioside GD3 levels were increased over those in age-matched untreated GM1 mice (Tables S4, S5, S6, S7, S8, S9, S10, S11), and in some instances close to levels found in HZ mice (e.g. Bs+ScS and cerebellum 4 months – Tables S5 and S8).

Intracranial AAV treatment had no other major effect on the distribution or content of the other individual gangliosides analyzed.

### Sustained reduction in GA1 glycosphingolipid in the CNS of AAV-treated GM1 gangliosidosis mice

**Figure 4:**
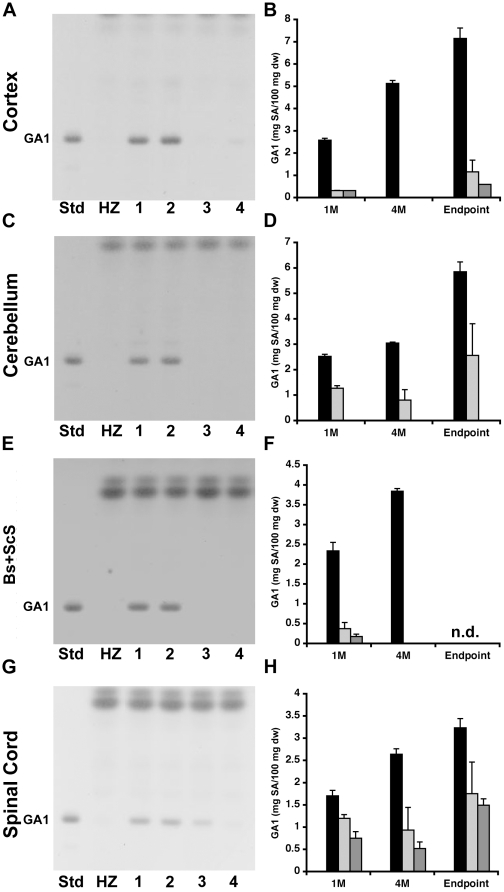
Biochemical quantification of GA1 content in the CNS of AAV-treated GM1 mice by HPTLC. GA1 isolated from (**A, B**) cortex, (**B, C**) cerebellum, (**D, E**) brainstem + subcortical structures (Bs+ScS), and (**F, G**) spinal cord of heterozygote (HZ, white bars*), untreated GM1 (1, black bars), PBS-treated GM1 (2), AAV-T GM1 (3, light gray bars), and AAV-TC GM1 (4, dark gray bars) mice. (**A, C, E, G**) Representative chromatograms for 4 months post-injection are shown. The amount of sample spotted per lane was approximately equivalent to 0.2 mg tissue dry weight. (**B, D, F, H**) Mean concentration of GA1 glycosphingolipid represented as mg of GA1/100 mg dry tissue weight (dw) for each region of the CNS at 1 month (1M), 4 months (4M) post-injection, and at the humane or experimental endpoint (Endpoint) are shown. Error bars represent 1 SEM. n.d. – not determined; * White bars = 0.

We analyzed the same regions of the CNS for all groups and time points for GA1 content because of the concomitant accumulation of this GSL in GM1 mice [6] (Fig. 4). As expected, GA1 levels were significantly reduced in all CNS regions and time points analyzed (p<0.01) for both groups of AAV-treated GM1 mice (light and dark gray bars in Fig. 4) compared to age matched untreated GM1 mice (black bars in Fig. 4). In AAV-T GM1 mice the lowest levels of GA1 were achieved at 4 months post-injection (Fig. 4B, D, H–4M light gray bars), but at endpoint, the GA1 content had increased to 1.2±0.52, 2.56±1.24, and 1.88±0.71 mg/100 mg dry tissue weight in cortex, cerebellum, and spinal cord, respectively (Fig. 4B, D, H – Endpoint light gray bars). Also in AAV-TC GM1 mice, the lowest levels of GA1 were achieved at 4 months post-injection (Fig. 4B, D, H–4M; dark gray bars = 0), albeit it was present in the spinal cord (Fig. 4H–4M; dark gray bar). At endpoint (52 weeks of age), GA1 remained undetectable in the cerebellum (Fig. 4C – Endpoint; dark gray bar = 0), but had increased to 0.59±0.01, and 1.49±0.14 mg/100 mg dry tissue weight in both the cortex, and spinal cord (Fig. 4B, G – Endpoint; dark gray bars). In untreated GM1 mice at the humane endpoint, GA1 levels were 7.15±0.47, 5.84±0.39, 3.23±0.21 mg/100 mg dry tissue weight in cortex, cerebellum, and spinal cord, respectively (Fig. 4B, D, and H – Endpoint; black bars).

### Cerebrosides are partially restored in the cortex of AAV-treated adult GM1 Mice

Cerebrosides are myelin-enriched lipids, and changes in their content in the CNS correlate with relative amount of myelin [39], [40]. The concentration of cortical cerebrosides for all groups at 1 and 4 months post-injection are shown in Table 1. In naïve and PBS-treated GM1 mice there was a statistically significant decrease in concentration of cerebrosides for both time points compared to HZ mice (p<0.05). This is consistent with previous findings of reduced myelin in animal models and in patients with GM1-gangliosidosis [41], [42], [43]. The concentration of cerebrosides in both AAV-treated groups at 1 month post-injection was comparable to that in naïve and PBS-treated GM1 mice. At 180 days of age, or 4 months post-injection, the concentration of cerebrosides was significantly greater in AAV-T GM1 mice than in naïve or PBS-treated GM1 mice. In AAV-TC GM1 mice the concentration of cerebrosides remained stable between the two time points, but below the levels in HZ control mice.

**Table 1:** Concentration of cortical cerebrosides in adult GM1 Mice^a^.

Genotype	Treatment	90 days of age	180 days of age
βgal+/−	-	2.43±0.14	2.54±0.14
βgal−/−	-	1.45±0.15#	1.39±0.07#
βgal−/−	PBS	1.40±0.25#	1.25±0.05#
βgal−/−	AAV-T	1.59±0.10	2.06±0.27*
βgal−/−	AAV-TC	1.73±0.16	1.79±0.19

a Concentration expressed as mg/100 mg dry weight. Values represent mean ± SEM, where 3 independent samples were analyzed per group.

# Indicates that the value is significantly different from that in untreated βgal+/− mice at P<0.05.

*Indicates that the value is significantly different from that in untreated βgal−/− mice at P<0.01.

### Effect of AAV treatment on disease marker gene expression

**Figure 5:**
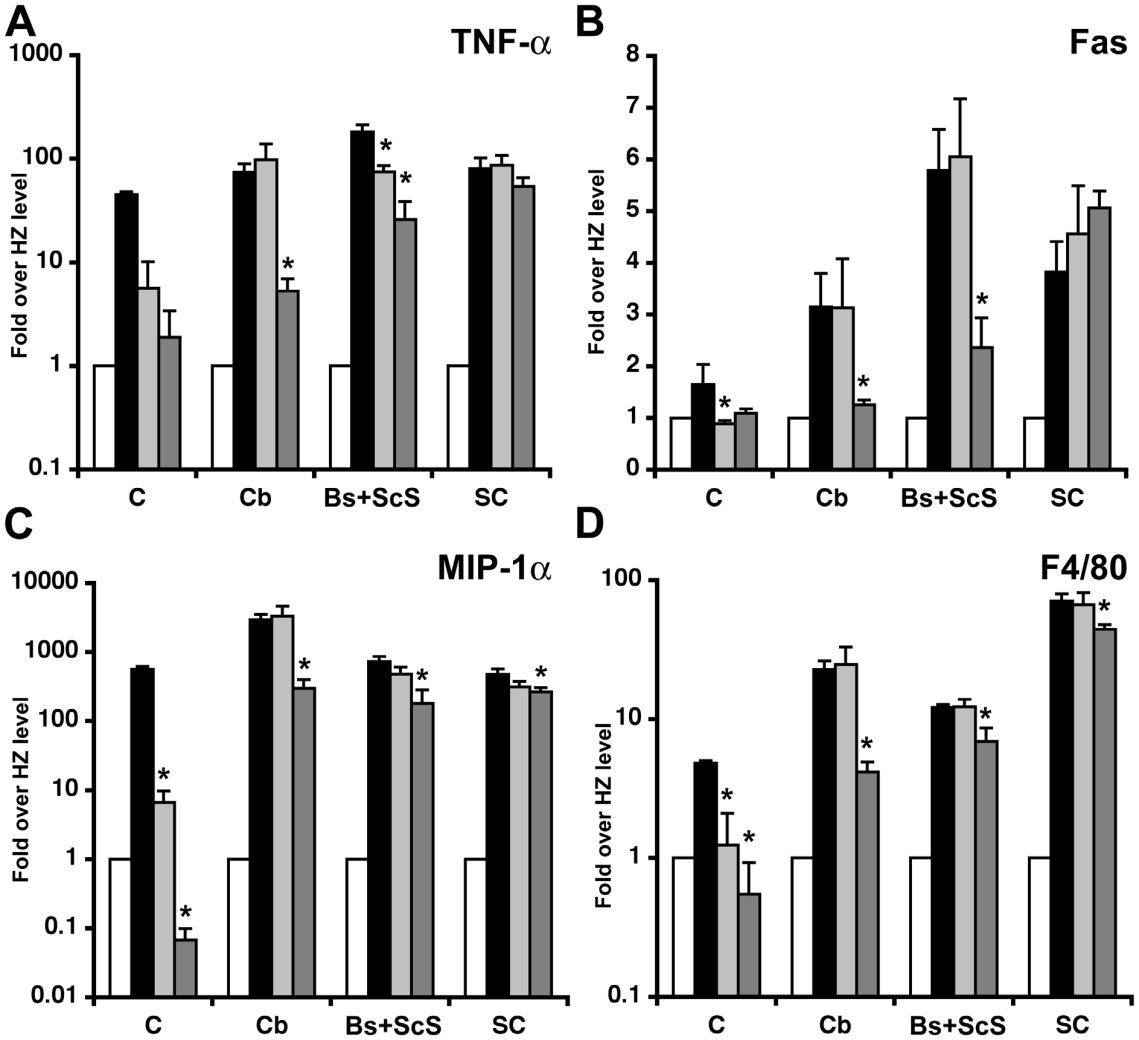
Disease marker gene expression in the CNS of AAV-treated GM1 mice. Untreated GM1 mice (black bars), and AAV-T GM1 mice (light gray bars) were sacrificed at the humane endpoint defined by >20% loss in body weight, or at 52 weeks of age for AAV-TC GM1 mice (dark gray bars), and HZ mice (white bars). Total RNA was isolated from cerebrum (c), cerebellum (Cb), brainstem+subcortical structures (Bs+ScS), and spinal cord (sc) and used for real-time PCR quantification of TNF-α (**A**), Fas (**B**), MIP-1α (**C**), and F4/80 (**D**) expression levels. Average fold induction over normal (HZ levels) was calculated for each tissue. Error bars correspond to 1 SEM. * indicates statistical significance with a p-value<0.05 in Student's t-test.

Neurodegeneration in GM1 mice was shown to be the result of GM1-mediated activation of both the UPR and the mitochondrial apoptotic pathway [10], [11]. Neuronal cell death elicits a neuroinflammatory process characterized by up-regulation of cytokines, chemokines, inflammatory cell markers, and microglial activation [12], [13]. Thus we analyzed the mRNA expression levels of some disease marker genes associated with inflammation, namely TNF-α, Fas, MIP-1α, F4/80 in the cortex, cerebellum, Bs+ScS, and spinal cord of mice sacrificed at the humane endpoint (untreated GM1 mice, PBS-treated GM1 mice, AAV-T GM1 mice), or 52 weeks of age (AAV-TC GM1 mice, and heterozygote mice) (Fig. 5). As expected most marker genes were elevated in untreated GM1 mice compared to naïve heterozygote mice, and in some instances such as MIP-1α by more than 100-fold (Fig. 5C, black bars). In AAV-T and AAV-TC GM1 mice there were statistically significant decreases in expression levels in some of the structures analyzed (asterisks in Fig. 5, p<0.05), but seldom to levels comparable to those in heterozygote control mice.

### Effect of AAV treatment on motor performance of GM1 mice

**Figure 6:**
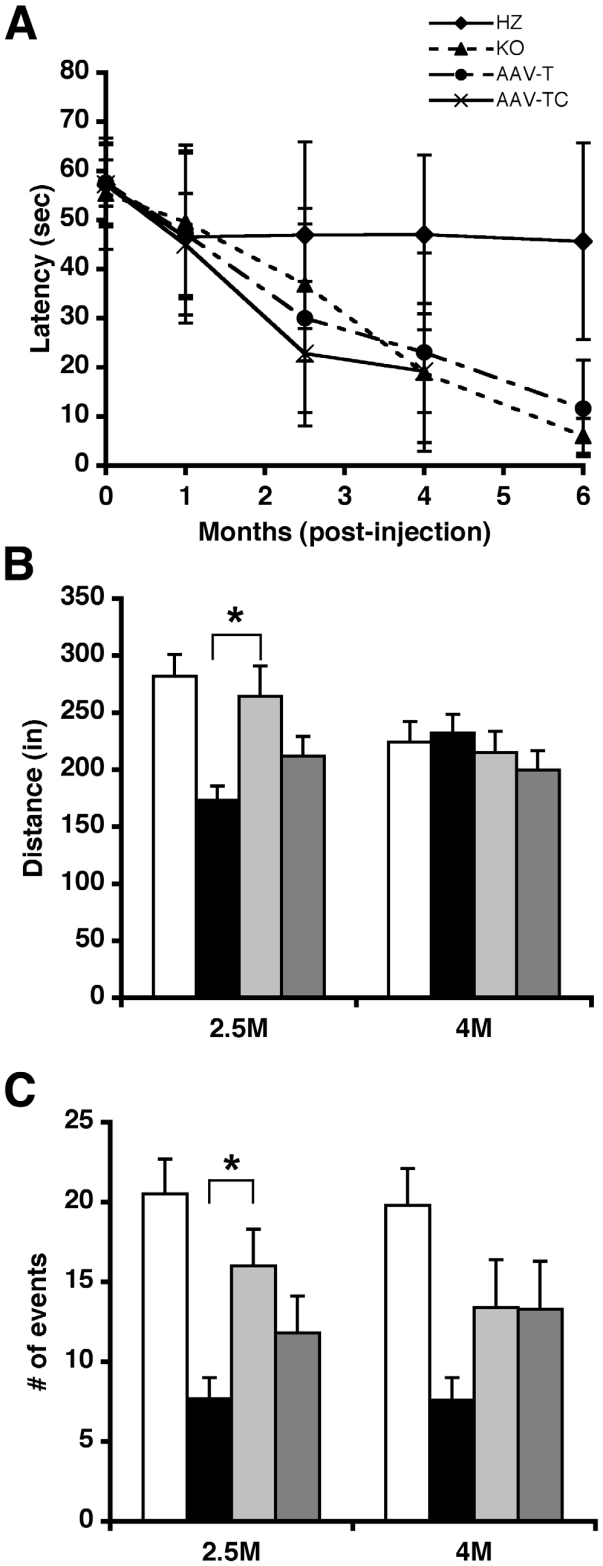
Effect of AAV-treatment on motor performance of GM1 mice. (**A**) Rotarod testing was performed prior to injection (0 months), and then at 1, 2.5, 4, and 6 months post-injection in AAV-T GM1 mice (•), AAV-TC GM1 (**✗**), untreated GM1 (▴), and HZ mice (⧫). Open-field testing measured (**B**) locomotor and (**C**) rearing activity at 2.5 (2.5M) and 4 (4M) months post-injection in HZ (white bars), untreated GM1 (black bars), AAV-T GM1 (light gray bars), and AAV-TC GM1 (dark gray bars) mice. Group sizes: n = 20–24 for 0 and 1 month time points; n = 14–18 for 2.5 and 4 month time points; n = 10–12 for 6 month time point. Graphs represent the mean for each group at the specified time point. Error bars correspond to 1 SEM. * p<0.05 in paired Student's t-test.

We used rotarod and open field tests to assess the motor performance of AAV-treated GM1 and control mice at different time points after injection (Fig. 6). Rotarod testing of all animals prior to intracranial injection of AAV vector showed comparable performance for all groups of mice (Fig. 6A). Mice were subsequently tested at 1, 2.5, 4, and 6 months post-injection. The performance of either group of AAV-treated GM1 mice (AAV-T and AAV-TC) declined over time, and was indistinguishable from that of control untreated GM1 mice (Fig. 6A). The performance of HZ control mice remained essentially stable for the duration of the experiment. Open-field testing was performed at 2.5 and 4 months post-injection to measure locomotor (Fig. 6B) and rearing activity (Fig. 6C). At 2.5 months post-injection the locomotor activity of both groups of AAV-treated GM1 mice was greater than untreated GM1 controls (Fig. 6B, 2.5M - black bar), but statistical significance (p<0.05) was only achieved in AAV-T GM1 mice (Fig. 6B, 2.5M – light gray bar). By 4 months post-injection the locomotor activity of AAV-treated GM1 mice declined and was indistinguishable from untreated GM1 control mice (Fig. 6B, 4M). The locomotor activity of untreated GM1 mice increased between the two time points (Fig. 6B, black bars, p<0.01), while that of HZ controls decreased (Fig. 6B, white bars, p<0.05). Although the number of rearing events in AAV-treated GM1 mice (Fig. 6C, gray bars) was consistently higher than in untreated GM1 mice controls (Fig. 6C, black bars), this difference was significant only in AAV-T GM1 mice at 2.5 months post-injection (p<0.05).

### Effect of AAV treatment on Visual Evoked Potentials in GM1 mice

**Figure 7:**
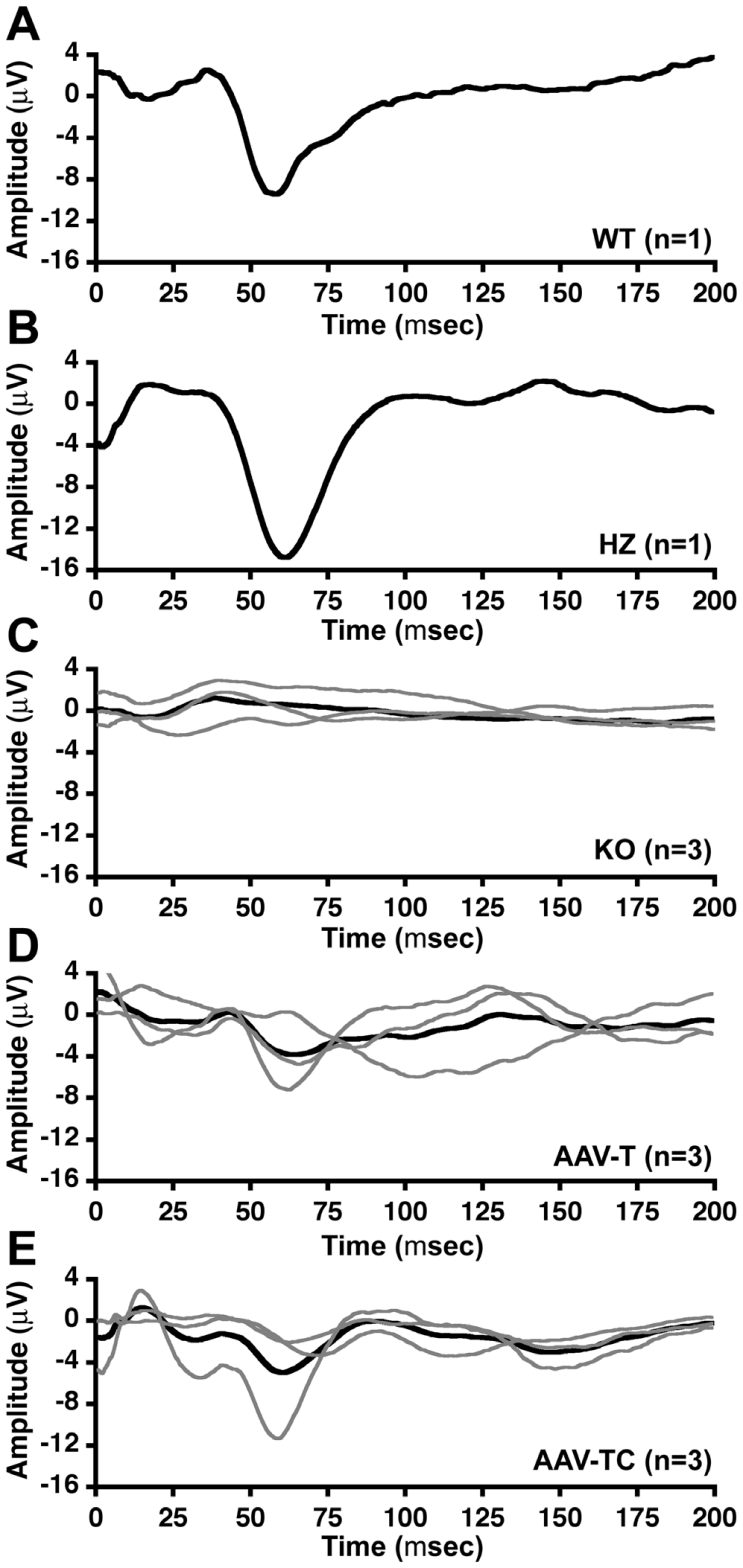
Effect of AAV treatment on visual function in GM1 mice. Visual evoked potentials were measured in (**A**) wild type, (**B**) HZ, (**C**) untreated GM1, (**D**) AAV-T GM1, and (**E**) AAV-TC GM1 mice. Group sizes are indicated on the graphs. (**C–E**) Gray lines show the results for each mouse in the group. Black lines represent the group average.

**Figure 8:**
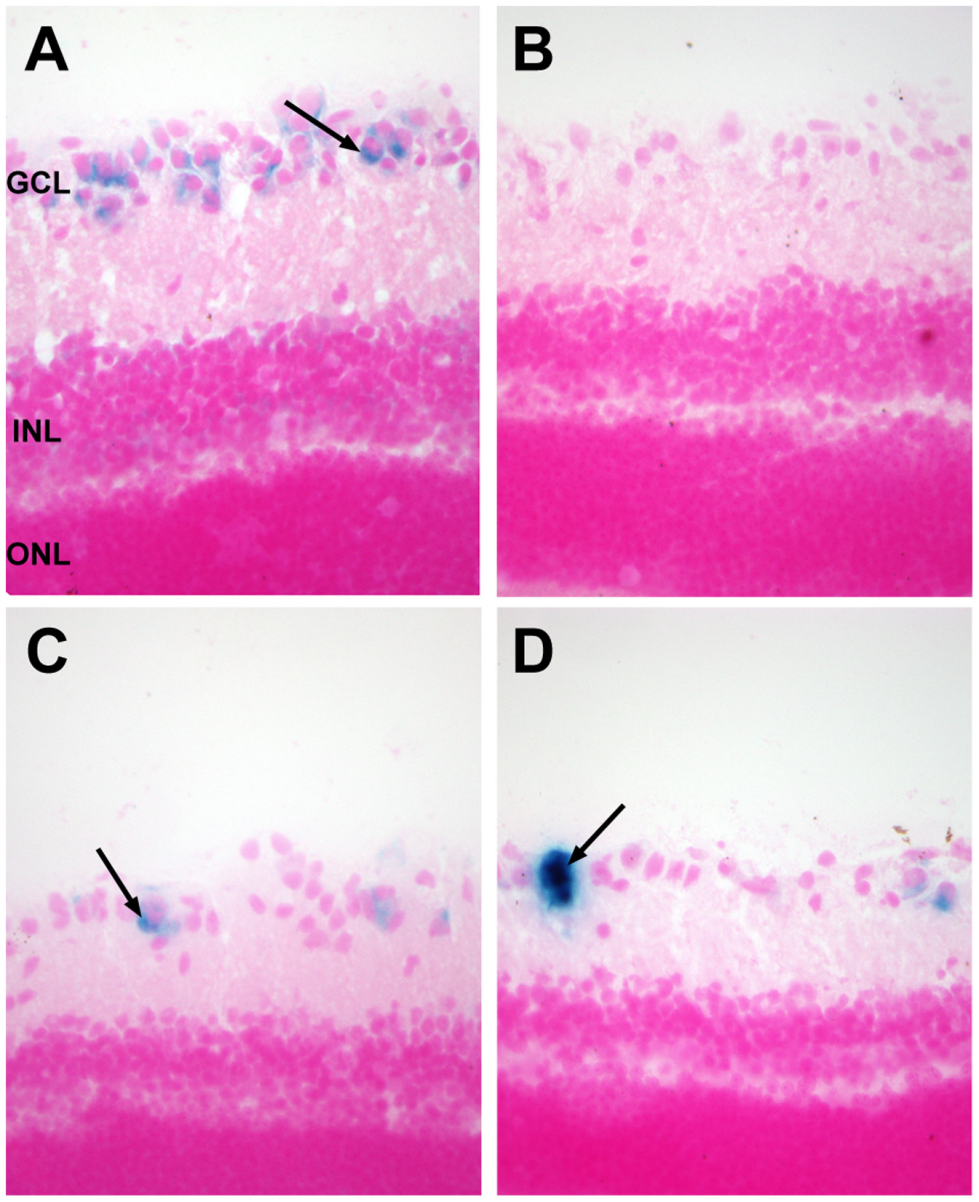
Presence of β-galactosidase activity in the retina of AAV-treated GM1-gangliosidosis mice. The eye was collected at the humane endpoint or 1 year of age, and βgal activity assessed by X-gal staining at pH 5.0 of histological sections from: (**A**) Heterozygote mice; (**B**) Untreated GM1 mice; (**C**) GM1 mice injected bilaterally in the thalamus (AAV-T group); (**D**) GM1 mice injected bilaterally in the thalamus and deep cerebellar nuclei (AAV-TC group). Sections were counterstained with Nuclear Fast Red reagent. Abbreviations: GCL- ganglion cell layer; INL – inner nuclear layer; ONL – outer nuclear layer. Magnification – 200×.

GM1 mice older than 6 months display visual abnormalities characterized by normal electroretinograms but subnormal visually evoked potentials (VEP) [28]. We analyzed VEPs in AAV-treated GM1 mice at 10–11 months of age, and age matched untreated control HZ mice (Fig. 7). Untreated GM1 mice were analyzed at 7–8 months of age, and presented subnormal VEPs (Fig. 7C) compared to wild type (Fig. 7A) and HZ (Fig. 7B) mice. AAV-treated GM1 mice showed some response to the visual stimulus (Fig. 7D, E), albeit with considerable variability among animals within each group (gray lines in Fig. 7D, E represent the VEP of each individual animal in the group). The VEP data also show, on average, normal negative peak implicit time (50–75 msec) for AAV-treated mice. These data suggest that AAV-treated GM1 mice retained some degree of visual functionality past 6 months of age. Histological analysis of the eye at the humane endpoint (untreated GM1 and AAV-T GM1 mice), or 1 year of age (heterozygote mice, and AAV-TC GM1 mice) showed evidence of some βgal activity in the retinal ganglion cell layer (GCL) in both groups of AAV-injected GM1 mice compared to no detectable activity in the retinas of untreated GM1 mice (Fig. 8).

### Effect of AAV treatment on survival

**Figure 9:**
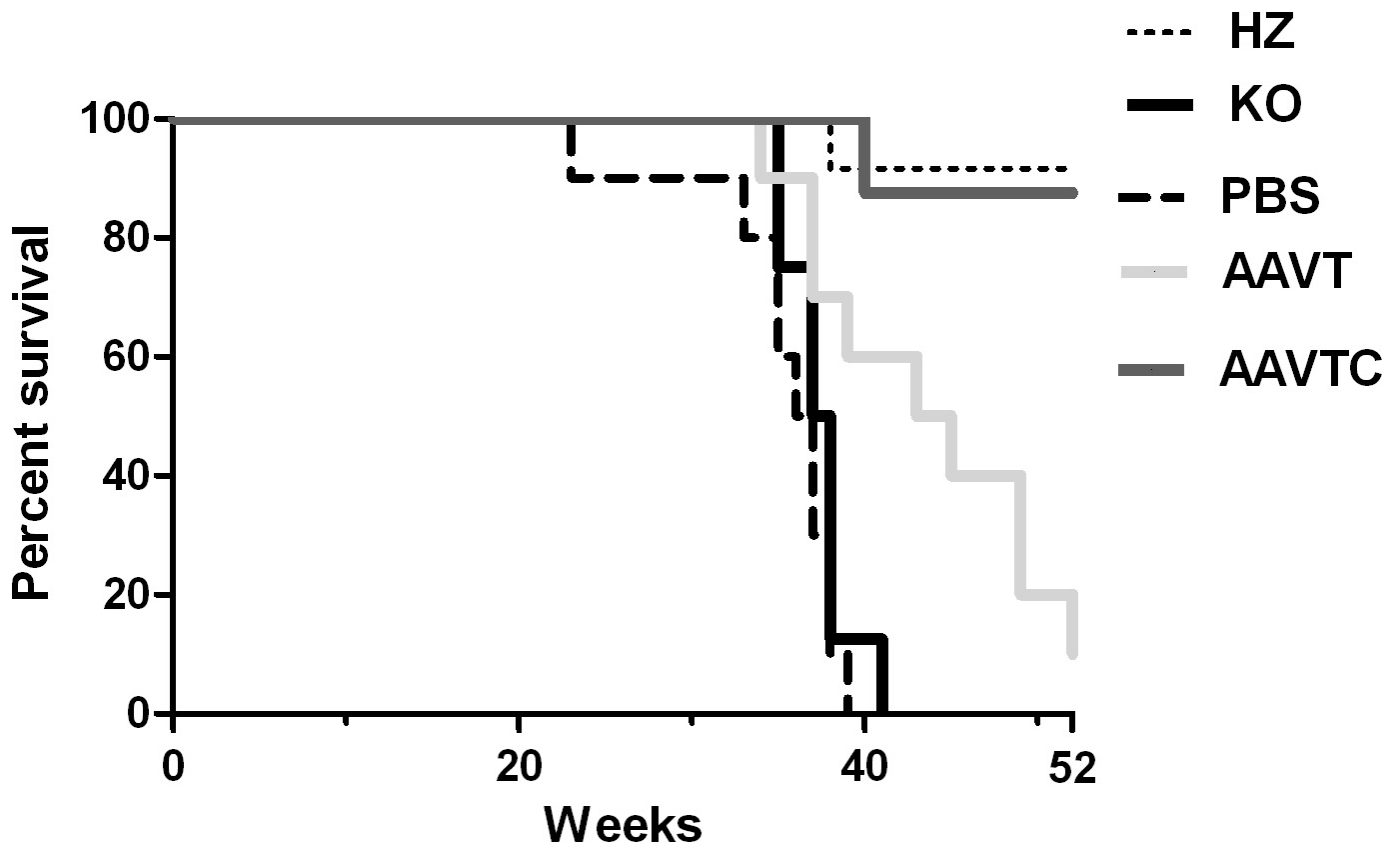
Kaplan-Meier survival analysis of AAV-treated GM1 mice. Untreated GM1 (KO, black line), PBS-treated GM1 (PBS, bold dashed line), heterozygote (HZ, dashed line), AAV-T GM1 (light gray line), and AAV-TC GM1 (dark gray line) mice were allowed to survive until the humane endpoint defined by >20% body weight loss, or 52 weeks of age. N = 8–12 per group.

In addition to sacrificing mice at different time points after treatment for biochemical and histological analyses, we allowed a subset of animals from each group (n = 8–12 animals per group) to survive until the humane endpoint defined by >20% loss in body weight. The median survival for untreated and PBS-injected GM1 mice was 38 and 37 weeks post-treatment, respectively (Fig. 9), while the median survival time for AAV-T GM1 mice was significantly increased to 45 weeks (p<0.05, Log-Rank test). All but one AAV-TC GM1 mouse (n = 8) survived until the 52-week experimental endpoint (p<0.001, Log-rank test), despite their poor performance in motor tasks. Although these animals never reached the humane endpoint as defined above, they displayed signs of disease such as tremor, and difficulty in moving the hind limbs at the experimental endpoint.

## Discussion

Previously, we had shown that a single AAV injection into the cerebral lateral ventricles of neonatal GM1 mice was sufficient to correct the neurochemical phenotype [17]. In this study, we examined the therapeutic efficacy of an AAV2/1 vector encoding mouse βgal infused bilaterally into the thalamus, or thalamus and deep cerebellar nuclei of adult GM1 mice. This intraparenchymal infusion approach led to sustained high-level expression of βgal in many of the CNS structures analyzed, and dramatic reductions in GM1-ganglioside and GA1 content. Surprisingly the motor performance of AAV-treated GM1 mice declined over time at a rate similar to that of untreated GM1 mice. Nonetheless, the median survival of both groups of AAV-treated mice was significantly increased compared to untreated GM1 mice.

Our results show that thalamic delivery of an AAV2/1 vector encoding mouse βgal is an effective approach to achieve widespread distribution of therapeutic levels of this enzyme in the adult GM1 mouse brain. The distribution of βgal in the brain was uneven with anterior parts of the brain receiving less enzyme than posterior regions closer to the injection site. The simplest explanation is that enzyme diffusion limits its distribution to areas near the injection site. However we have shown that βgal, like many other lysosomal enzymes [18], [20], can be transported by axonal retrograde transport from the site of injection [21]. Thus an alternative explanation is that the βgal distribution pattern observed here is a direct result of the tropism of AAV2/1 vector for a subset of thalamic nuclei, namely dorsal and lateral nuclei. The faint ISH signal present in the cerebral cortex (Fig. 1G–I) could be due to axonal transport of vector-derived mRNA in thalamic neurons that project to the cerebral cortex, similar to our previous observation in the hippocampal system [21]. Alternatively, it could be due to leakage of vector along the needle track with subsequent transduction of cortical cells. This could explain the intense staining observed in these cortical regions. Additional experiments will be necessary to determine whether more widespread transduction of thalamic nuclei leads to wider distribution of βgal in the brain. Others have also injected AAV vectors encoding lysosomal enzymes into the adult thalamus in mouse models of other LSDs, but it has been done in combination with several other targets, and thus it is difficult to assess the contribution of thalamic transduction to the overall pattern of enzyme distribution in the CNS [37], [44], [45]. Recently, Kells and colleagues showed that thalamic infusion of an AAV2 vector encoding GDNF resulted in extensive distribution of this growth factor to the frontal cortex [46]. These results support the notion that AAV-mediated genetic modification of thalamus may indeed be an effective approach to distribute other secreted therapeutic proteins to large regions of the brain.

The main finding from our studies is that bilateral injections of AAV vector in adult GM1 mice lead to sustained reduction of GM1-ganglioside and GA1 content to nearly normal levels in most CNS structures analyzed. The spinal cord was an exception, however, as GM1 levels were reduced to only ∼50% of the levels found in age-matched untreated GM1 mice. Both groups of AAV-treated mice survived significantly longer than untreated GM1 mice, with the group receiving bilateral injection of AAV vector in the thalamus and DCN (AAV-TC group) surviving until 1 year of age. Nonetheless the motor performance of both groups of AAV-treated GM1 mice declined over time and was indistinguishable from that of age-matched untreated GM1 mice. According to our neurochemical analysis, combined bilateral injection into thalamus and DCN (AAV-TC group) is effective in restoring GM1-ganglioside level to normal and eliminating GA1 glycosphingolipid in the cerebellum, while bilateral thalamic injections (AAV-T group) lead to partial correction (Fig. 3D, light gray bars), despite achieving enzymatic activity levels comparable to those in cerebellum of HZ mice (Fig. 1D, light gray bars) (This apparent paradox is addressed below). The effect on lipid content in other CNS structures is comparable in both treatment groups. The difference in median survival between AAV-T and AAV-TC GM1 mice suggests that disease progression in the cerebellum is a contributing factor to the phenotype in GM1 mice. Previous studies have shown that the earliest and most profound changes in neuroinflammatory markers and cytokine production are found in cerebellum, brainstem and spinal cord [12], [13]. The unexpected finding in this study was that GM1-ganglioside and GA1 levels in the spinal cord were essentially the same in both groups of AAV-treated mice. βgal activity in the spinal cord of both groups of AAV-treated GM1 mice was comparable at all time points analyzed at approximately 50% of enzymatic activity in HZ spinal cord (same observation as in the cerebellum of AAV-T GM1 mice). This level of enzymatic activity should be sufficient to completely correct lysosomal storage in the spinal cord. This apparent paradox may be explained by transport of βgal from injected structures in the brain to a relatively small subset of cells in the spinal cord where βgal activity reaches high levels and skews the total enzymatic activity in the tissue. The fact that we do not observe normalization of GM1-ganglioside level and elimination of GA1 glycosphingolipid suggests that secondary distribution of enzyme to other cells may be inefficient. Our present observation that injection of an AAV vector into DCN does not improve significantly lysosomal storage in the spinal cord is surprising. Previous studies have shown that AAV-mediated gene transfer to DCN in mice is an effective approach to supply lysosomal enzymes and growth factors to the spinal cord [23], [24]. Moreover this target has also been utilized in several studies of AAV-mediated gene delivery to the adult brain in mouse models of other LSDs, but also in the context of multi-target injections [37], [44], [47], and thus difficult to discern the contribution of AAV-transduced DCN to the overall therapeutic effect. Several explanations are possible for our results: 1) Stereotaxic injections missed the intended DCN target, and transduction of other cerebellar regions is sufficient to supply the cerebellum with therapeutic levels of βgal, but inadequate for axonal transport of enzyme to the spinal cord; 2) differences in vector preparation/purification method that could affect its tropism, similar to the effect reported for other AAV serotypes [48]; 3) severity of spinal cord involvement may be disease-specific, and in GM1 mice it appears to be one of the first structures in the CNS where alterations are detected [12], [13]; 4) higher levels of βgal may be necessary in the spinal cord of GM1 mice to achieve therapeutic correction compared to the amount of enzyme necessary in other mouse models of LSDs. Although we documented nearly normal GM1-ganglioside levels and complete elimination of GA1 in cerebrum (cortex, brainstem and subcortical structures) and cerebellum of AAV-TC GM1 mice at 4 months post-injection (5.5 to 6 months of age), these glycosphingolipids remained elevated in the spinal cord (2.2-fold above normal). Since motor performance was essentially indistinguishable from age matched untreated GM1 mice, disease progression in the spinal may be responsible for the progressive decline in motor performance of AAV-treated GM1 mice in the present study.

Myelin loss has been reported in patients and animal models of GM1- gangliosidosis [41], [42]. Previously we have shown that intracerebroventricular injection of an AAV2/1-CBA-βgal vector in neonatal GM1 mice restored cerebrosides, which are myelin-enriched lipids, to normal levels in the cerebrum at 3 months of age [17]. The concentration of cerebrosides is directly proportional to the amount of myelin and has been used as a myelin marker in remyelination studies in mice [49], [50], [51]. Our results further document the presence of a biochemical alteration in GM1 mouse myelin, and suggest that AAV-treatment partially corrects it (Table 1). Previous studies have shown that the timing of intervention is a critical parameter to prevent axonal degeneration in classical late infantile neuronal ceroid lipofuscinosis mice, where early treatment extends considerably the longevity of AAV-treated mice [44]. We have performed additional studies in 4 week-old GM1 mice injected bilaterally in the thalamus and DCN with the same dose of AAV2/1-CBA-mβgal vector used in this study (with and without i.v. infusion of liver-specific AAV vector) to test this possibility, but did not observe any improvement in motor performance compared to age matched untreated GM1 mice (data not shown).

A potentially important observation in this study was the apparent preservation of some visual function in AAV-treated GM1 mice beyond 6 months of age, while age-matched untreated GM1 control mice did not respond to visual stimuli, as previously described [28]. Also the observation of normal peak implicit times with reduced amplitudes in the averaged responses in AAV-treated mice suggests that some (but not all) cells in the retina may have retained normal function. Since retinal ganglion cells project to the lateral geniculate nucleus in the thalamus, and this region appeared to express high levels of βgal (as indicated by some of the darkest X-gal staining anywhere in the brain – See Fig. 1), it is possible that some enzyme and/or AAV vector may have been transported via retrograde axonal transport to the retina. The histological evidence of βgal activity in the retinal ganglion cell layer of AAV-treated GM1 mice supports this notion, and could be the basis for preservation of some visual function in these mice. The effectiveness of transport of lysosomal enzymes and/or AAV vector from the thalamus to the eye requires additional experiments to characterize visual function in detail (electroretinograms, VEP and acuity) over time, and correlate it to GM1-ganglioside levels and histopathology in the retina.

Although it is possible that storage in other structures in the CNS, or remaining myelin deficits, may be involved in the long-term phenotype observed in AAV-treated GM1 mice, the symptoms that were displayed throughout the experiment are consistent with spinal cord involvement. Thus we hypothesize that to achieve long-term survival of GM1 mice with stable or improved motor performance, it will be necessary to devise a strategy to effectively address disease progression in the spinal cord. Several recent studies have shown that infusion of recombinant lysosomal enzymes into the cerebral spinal fluid via the lateral ventricles or intrathecally, is an effective approach to reach therapeutic levels throughout the CNS [52], [53], [54]. Also intracerebroventricular and intrathecal infusion of AAV vectors appear to be effective in delivering therapeutic levels of lysosomal enzymes to many regions of the CNS in various mouse LSD models [55], [56]. Combination of bilateral thalamic injections with one of these modalities may be an effective way to achieve complete correction throughout the CNS. Other targets in the CNS such as the ventral tegmental area (VTA) [22] and external capsule (EC) [57] appear to be highly effective to achieve widespread distribution of lysosomal enzymes in the brain after a single injection of an AAV vector. Rigorous studies will be necessary to compare the effectiveness of different AAV vector engineered enzyme producing centers in the brain (thalamus, VTA, EC) to achieve global distribution of those enzymes throughout the adult GM1 mouse CNS. To our knowledge this is the first study demonstrating the potential of thalamic gene delivery to achieve global distribution of a lysosomal enzyme throughout the mouse cerebrum. Our studies suggest that the thalamus can be used as a central node in a ‘built-in’ network for widespread distribution of lysosomal enzymes, and possibly other secreted therapeutic proteins throughout the cerebrum.

## Supporting Information

Table S1 Cortical ganglioside distribution in adult GM1 Mice, 1 month post AAV ic injection.
(0.03 MB XLS)

Table S2 Cortical ganglioside distribution in adult GM1 mice, 4 months post AAV ic injection.
(0.03 MB XLS)

Table S3 Cortical ganglioside distribution in adult GM1 mice, at endpoint or 10 months post AAV ic injection.
(0.03 MB XLS)

Table S4 Brainstem+subcortical structures ganglioside distribution in adult GM1 mice, 1 month post AAV ic injection.
(0.03 MB XLS)

Table S5 Brainstem+subcortical structures ganglioside distribution in adult GM1 mice, 4 months post AAV ic injection.
(0.03 MB XLS)

Table S6 Cerebellar ganglioside distribution in adult GM1 mice, 1 month post AAV ic injection.
(0.03 MB XLS)

Table S7 Cerebellar ganglioside distribution in adult GM1 mice, 4 month post AAV ic injection.
(0.03 MB XLS)

Table S8 Cerebellar ganglioside distribution in adult GM1 mice, at endpoint or 10 months post AAV ic injection.
(0.03 MB XLS)

Table S9 Spinal Cord ganglioside distribution in adult GM1 mice, 1 month post AAV in injection.
(0.03 MB XLS)

Table S10 Spinal Cord ganglioside distribution in adult GM1 mice, 4 month post AAV ic injection.
(0.03 MB XLS)

Table S11 Spinal Cord ganglioside distribution in adult GM1 mice, at endpoing or 10 month post AAV ic injection.
(0.03 MB XLS)
